# Vestibular rehabilitation ameliorates chronic dizziness through the SIRT1 axis

**DOI:** 10.3389/fnagi.2014.00027

**Published:** 2014-03-04

**Authors:** Chung-Lan Kao, Kun-Ling Tsai, Yuan-Yang Cheng, Chia-Hua Kuo, Shin-Da Lee, Rai-Chi Chan

**Affiliations:** ^1^Department of Physical Medicine and Rehabilitation, Taipei Veterans General HospitalTaipei, Taiwan; ^2^School of Medicine, National Yang-Ming UniversityTaipei, Taiwan; ^3^Department of Physical Therapy and Assistive Technology, School of Biomedical Science and Engineering, National Yang-Ming UniversityTaipei, Taiwan; ^4^Department of Physical Medicine and Rehabilitation, Taichung Veterans General HospitalTaichung, Taiwan; ^5^Laboratory of Exercise Biochemistry, Department of Sport Sciences, University of TaipeiTaipei, Taiwan; ^6^Department of Physical Therapy, Graduate Institute of Rehabilitation Science, China Medical UniversityTaichung, Taiwan

**Keywords:** dizziness, SIRT1, exercise, oxidative stress, FoxO

## Abstract

Dizziness is a common clinical symptom frequently referred to general neurologists and practitioners. Exercise intervention, in the form of vestibular rehabilitation, is known as an effective clinical management for dizziness. This intervention is reported to have a functional role in correcting dizziness, improving gaze stability, retraining balance and gait, and enhancing physical fitness. Dizziness is known to be highly related to inflammation and oxidative stress. SIRT1 is a major molecule for the regulation of inflammation and mitigation of oxidative stress in chronic diseases such as atherosclerosis and chronic obstructive pulmonary disease. However, the bio-molecular roles of SIRT1 involved in the pathogenesis of dizziness are still largely unclear. In this study, a total of 30 subjects were recruited (15 patients with chronic dizziness, and 15 age/gender matched non-dizzy control subjects). The dizzy subjects group received 18 sessions of 30-min vestibular training. We found that the mRNA and protein expression levels of SIRT1 in the blood samples of chronic dizzy patients were repressed compared with those of healthy controls. After vestibular training, the dizzy patients had significant symptomatic improvements. The SIRT1 expression and its downstream genes (PPAR-γ and PGC-1α) were upregulated after vestibular exercises in dizzy subjects. Notably, the catalytic activity of SIRT1, NADPH and antioxidant enzyme activities were also activated in dizzy patients after vestibular training. Furthermore, vestibular exercise training reduced oxidative events and p53 expression in patients with dizziness. This study demonstrated that vestibular exercise training improved dizziness symptoms, and mechanisms for alleviation of chronic dizziness may partly involve the activation of the SIRT1 axis and the repression of redox status.

## INTRODUCTION

Dizziness is a common clinical symptom observed in internal medicine clinics (Bisdorff et al., 2009). Dizziness disorders can originate with or without a prior organic vestibular dysfunction (Schubert and Minor, 2004; Eckhardt-Henn et al., 2008). The prevalence and severity of dizziness has been reported to increase with age (Colledge et al., 1994). For patients over the age of 75 years, dizziness is the most common reason for visiting a doctor (Eaton and Roland, 2003). Dizziness disorders are highly correlated with phobic, panic, anxiety, depressive, dissociative or somatoform disorders (Dieterich and Eckhardt-Henn, 2004). Importantly, the histories from those patients with dizziness disorders point to dizziness disorders being an inflammatory disease, including peripheral labyrinthine abnormalities or inflammation of inner ears (Paparella et al., 1990). Studies have been conducted to examine the relationship between reactive oxygen species (ROS) and dizziness (Semaan et al., 2005). Results suggest that dizziness is positively related to oxidative stress. Treatment using antioxidants have shown remarkable improvement of uncomfortable symptoms in Meniere’s disease (Raponi et al., 2003; Takumida et al., 2003). Although details of the bio-molecular mechanism causing dizziness are still elusive, current evidence has pointed to a relationship between oxidative stress and vertigo (Raponi et al., 2003). Further research is still required to elucidate the molecular interactions of oxidative stress, dizziness and the effects of treatment.

To treat dizziness, vestibular rehabilitation remains the primary exercise treatment (Telian and Shepard, 1996). Vestibular rehabilitation is a program of exercises to promote central nervous system compensation; it is a process through which other non-vestibular sensory inputs can be enhanced to control an individuals’ equilibrium (Dominguez, 2005). Vestibular rehabilitation is reported to have a functional role in correcting dizziness symptoms, improving gaze stability, retraining balance and gait, and enhancing physical fitness (Luxon, 2004). Boyer et al. (2008) suggested that exercise training may provide positive feedback for patients suffering from vertigo and dizziness. Moreover, exercise training has long been proven to be effective in repressing pro-inflammatory responses, such as Aβ-induced inflammation of neurons, and mitigating the pro-inflammatory parameter in LPS-injected rats (Kang et al., 2013; Kim et al., 2013).

The most well-investigated human sirtuins family is sirtuin 1 (SIRT1). SIRT1 is an essential mediator of longevity in normal cells (Moreau et al., 2007). Previous studies have revealed that SIRT1 deacetylates both histone and non-histone substrates, indicating that SIRT1 may participate in a key function in cellular physiological processes, such as metabolism, cell degeneration, cell growth and cell survival (Sauve et al., 2006). SIRT1 plays a vital role in regulating inflammation events, much like the mitogen-activated protein kinase family (MAPKs) or NF-κB. SIRT1 knock-down promotes NF-κB activity and pro-inflammatory cytokine secretion (Milne and Denu, 2008; Yoshizaki et al., 2010). Moreover, SIRT1 deacetylates and mitigates the transcription activity of activator protein-1 (AP-1), thereby inhibiting expression of the pro-inflammatory gene cyclooxygenase-2 (COX-2; Gao and Ye, 2008). SIRT1 regulates longevity factors and several factors by deacetylation of Forkhead box (FOXO) family (Salminen and Kaarniranta, 2010), SIRT1 probably acts via different mechanisms to regulate age-related changes including increasing mitochondriogenesis via modulating PGC-1α deacetylation, repressing oxidative stress survival response via FOXO family, reducing apoptosis and proliferation caused by p53 deacetylation, and mitigating pro-inflammatory response via NF-κB activation (Yeung et al., 2004; Lavu et al., 2008).

Exercise has been believed to be an efficient non-pharmacological intervention for human health; moreover, many studies also have shown that exercise training improves human physiological function (De Angelis et al., 2002; Ghosh et al., 2005). Ljubicic et al. (2012) suggested that activation of AMPK positively regulates SIRT1 and PGC-1α expression in dystrophic skeletal muscle, thereby improving movement performance. Exercise has been considered a positive regulator in controlling SIRT1 expression (Williams and Gurd, 2012). Activation of SIRT1 may be an effective intervention to improve health. However, the effects of exercise training to regulate SIRT1 expression in patients with dizziness have not yet been reported. We hypothesized that the expression and catalytic activity of SIRT1 would be repressed and involved in the pathogenesis of patients with chronic dizziness. The present study aimed to examine whether exercise training would positively activate SIRT1 expression and SIRT1-related downstream target-genes in patients with chronic dizziness.

## MATERIALS AND METHODS

### SUBJECTS

Patients with chief complaints of dizziness for more than 1 year were recruited from the Department of Physical Medicine and Rehabilitation, Taipei Veterans General Hospital. The diagnosis was based on patients’ self-reported histories, the results of head thrust tests, horizontal and vertical head shaking nystagmus tests and caloric examinations (AIRSTAR, Micromedical Technologies, IL, USA). Age-matched subjects without history of dizziness or vertigo were recruited as controls. Exclusion criteria included subjects with benign paroxysmal positional vertigo and cerebrovascular diseases. All of the participants were asked to sign an informed consent form approved by Taipei Veterans General Hospital Institutional Review Board.

### EXERCISE PROTOCOL

Each subject with dizziness received an exercise training session 3 days a week for 6 weeks. Each training session lasted a total of 30 to 40 min. Each subject was asked to read a target 2 m in front of them at eye level. Training started with a horizontal head movement at a comfortable speed for each subject. The head movements then increased to at least 120° per second to achieve a therapeutic goal. The participants changed positions from sitting to standing and from standing on a stable floor to standing on an unstable cushion surface. For the strengthening and balance exercises, the subjects were asked to sit on a chair, raise both legs with full knee extension and hold for 30 s, rest for 10 s and repeat for a total of 10 min. Then, the subjects were asked to stand on one leg for 30 s, hold on to a chair to keep balance if necessary, rest for 20 s, change legs, and repeat the same procedure The exercise was repeated for 10 min (Herdman et al., 2001; Chen et al., 2012).

### CLINICAL ASSESSMENTS

The visual analog scale (VAS), the Dizziness Handicap Inventory (DHI; Jacobson and Newman, 1990) and the Tinetti Fall Risk Performance Scale (POMA; Tinetti, 1986) were used to obtain information of the self-perceived dizziness level and balance functions for the control subjects. These scales were also applied to the chronic dizzy subjects to establish a baseline comparison before and after 6 weeks of exercise training. The DHI is a validated 25-item questionnaire for evaluating individuals’ self-perceived levels of dizziness-related handicap. A higher DHI score indicates a greater level of handicap caused by dizziness. The POMA is a 2-point scale for evaluating static and dynamic functions. A higher POMA score indicates a better balance function.

### ISOLATION OF mRNA AND QUANTITATIVE REAL-TIME

#### PCR

Six milliliters of blood from each subject were collected (BD Vacutainer system, K2-EDTA tubes; BD Diagnostics, Franklin Lakes, NJ, USA). Total RNA was isolated with an RNeasy Plus mini kit (Qiagen, Hilden, Germany). After the RNA was isolated, its quality was assessed with an Experion Automated Electrophoresis Station (Bio-Rad). Oligonucleotides for SIRT1, PGC-1a, p53, FoxO1, FoxO3, and β-actin were designed using the computer software package Primer Express 2.0 (Applied Biosystems, Foster City, CA, USA). All of the oligonucleotides were synthesized by Invitrogen (Breda, The Netherlands). Oligonucleotide specificity was determined by a homology search within the human genome (BLAST, National Center for Biotechnology Information, Bethesda, MD, USA) and confirmed by dissociation curve analysis. The oligonucleotide sequences are shown in **Table 1**. PCR was performed with SYBR Green in an ABI 7000 sequence detection system (Applied Biosystems) according to the manufacturer’s guidelines. The thermal cycling conditions consisted of a pre-incubation lasted for 5 min at 95°C, followed by 40 cycles of denaturation for 15 s at 95°C, followed by annealing for 30 s at 60°C and extension for 30 s at 72°C, and a final extension for 10 min at 72°C. The relative expression of target mRNA to β-actin mRNA was calculated.

**Table 1:** The oligonucleotide sequences.

Gene	Sense	Anti-sense
SIRT1	5′-TGTGGTAGAGCTTGCATTGATCTT-3′	5′-GGCCTGTTGCTCTCCTCAT-3′
p53	5′-GCCCACTTCACCGTACTAA-3′	5′-TGGTTTCAAGGCCAGATGT-3′
PGC-1	5′-CCGCACGCACCG AAA-3′	5′-TCGTGCTGATATTCCTCGTAGCT-3′
PPAR-y	5′-AGTGTGAATTACAGC AAATCTCTGTTTT-3′	5′-GCACCATGCTCTGGGTCA A-3′
FoxOl	5′-ATGGTCAAGAGCGTGCCC-3′	5′-GATTGAGCATCCACCAAG-3′
FoxO3	5′-TCTCCCGTCAGCCAGTCTAT-3′	5′-AGTCACTGGGGA ACTTGTCG-3′
β -actin	5′-CGGGAAATCGTGCGTGAC-3′	5′-TGCCCAGGAAGGAAGGCT-3′

### IMMUNOBLOTTING

Plasma was collected from whole blood sample after 2500 × *g* centrifuge at 4°C for 10 min, and then was separated by electrophoresis on SDS-polyacrylamide gel. After the proteins had been transferred onto a PVDF membrane (Millipore, Bedford, MA, USA), the blot was incubated with blocking buffer for 1 h at room temperature and then probed with SIRT1 primary antibody overnight at 4°C, followed by incubation with horseradish peroxidase-conjugated secondary antibody for 1 h. To control equal loading of total protein in all lanes, blots were stained with mouse anti β-actin antibody. Bound immunoproteins were detected by an enhancer chemiluminescent assay (ECL; Amersham, Berkshire, UK). The intensities were quantified by densitometric analysis (Digital Protein DNA Imagineware, Huntington Station, NY, USA).

### SIRT1 ACTIVITY ASSAY

SIRT1 activity in the samples was measured using the Cyclex SIRT1 Deacetylase Fluorimetric Assay Kit according to the manufacturer’s protocol (CycLex Ltd., Nagano, Japan) as previously described (Csiszar et al., 2009). In brief, upon NAD-dependent deacetylation of the specific substrate by SIRT1, the fluoro-substrate peptide is cleaved by a lysyl endopeptidase, separating the quencher from the fluorophore. By measuring time-dependent changes in fluorescence intensity produced by the fluorophore, specific activities of SIRT1 could be assessed (Kao et al., 2010a).

## NAD+/NADH MEASUREMENT

To determine the NAD+/NADH ratio, plasma was collected from whole blood after 2500 × *g* centrifuge at 4°C for 10 min. NAD+/NADH assay (abcan: ab65348) in the plasma was determined via an enzymatic assay method using a commercial kit according to the manufacturer’s instructions. The concentration of hydrogen peroxide was converted to μM. The concentrations of NAD+ and NADH were expressed in nmol per mg protein based on standard NADH readings.

### ANTIOXIDANT ENZYME ACTIVITY MEASUREMENT

To determine the antioxidant enzyme activity, plasma was collected from whole blood after 2500 × *g* centrifuge at 4°C for 10 min. SOD (Cell Biolabs, STA-340) and catalase activity (Cell Biolabs, STA-341) in the plasma was determined via an enzymatic assay method using a commercial kit according to the manufacturer’s instructions. Enzyme activity was converted to units per milligram of protein.

### HYDROGEN PEROXIDE MEASUREMENT

To determine the concentration of hydrogen peroxide, plasma was collected from whole blood after 2500 × *g* centrifuge at 4°C for 10 min. Hydrogen peroxide (Cell Biolabs, STA-344) in the plasma was determined via an enzymatic assay method using a commercial kit according to the manufacturer’s instructions. The concentration of hydrogen peroxide was converted to μM.

### MALONDIALDEHYDE ASSAY

Plasma was collected from whole blood after 2500 × *g* centrifuge at 4°C for 10 min. The plasma was then suspended in 130 mM KCl and 50 mM PBS containing 0.1 mL of 0.1 M dithiothreitol (DTT) and centrifuged at 20,000 *g* for 15 min at 4°C. The supernatant was taken for biochemical estimation. The level of lipid peroxidation was determined by analyzing TBARS, as previously described (Niehaus and Samuelsson, 1968). The pink-colored chromogen formed by the reaction of 2-TBA with the breakdown products of lipid peroxidation was measured.

### STATISTICAL ANALYSES

Data were expressed as the means ± standard deviation. Student* t-*test was used to analyze the evaluation of clinical assessments obtained before and after vestibular training in subjects with dizziness. The experimental results were analyzed with one-way ANOVA, and Bonferroni’s *post hoc* test was performed to compare the hematological mRNA level and plasma parameters. The criterion for significance was *p* < 0.05.

## RESULTS

### SUBJECT CHARACTERISTICS AND CLINICAL ASSESSMENT SCORES

To explore the differences of clinical manifestation between dizzy and non-dizzy individuals, 15 patients (mean age 70.73 ± 5.48 years, male:female = 8:7 dizzy group) who suffered from chronic dizziness (mean disease duration 3.2 ± 1.62 years; 12 patients with and 3 patients without a history of vestibular hypofunction) and 15 subjects (mean age 69.12 ± 3.62 years, male:female = 7:8 non-dizzy group) without a history of dizziness were enrolled in this study. No age or gender differences were found between groups (student *t*-test, *p* > 0.05). Clinical assessments were applied to evaluate the self-perceived sense of dizziness and balance function of the non-dizzy subjects on initial examinations. The mean VAS score was 0.40 ± 0.63. Total DHI score was 1.33 ± 1.80 and the POMA score was 26.27 ± 1.44. The same assessments were applied to the dizzy subjects before and after exercise training. The mean VAS score before exercise training for subjects in the dizzy group was 4.46 ± 3.24, the total DHI score was 35.07 ± 24.73 and the POMA score was 17.80 ± 3.86. Significant differences of clinical assessment scores were found between the dizzy and non-dizzy groups (*p* < 0.001). After 6 weeks of vestibular training, the VAS score improved to 3.46 ± 2.43 (*p* = 0.018) in dizzy group. The total DHI score changed to 24.53 ± 24.70 (*p* = 0.014), and the POMA score changed to 22.80 ± 2.80 (*p* < 0.0001). The clinical scores differed significantly before and after exercise training, suggesting that exercise intervention decreased the symptoms of dizziness and improved balance functions in subjects of the dizzy group.

### THE EXPRESSION LEVEL OF SIRT1 WAS ATTENUATED IN PATIENTS WITH CHRONIC DIZZINESS

**FIGURE 1:**
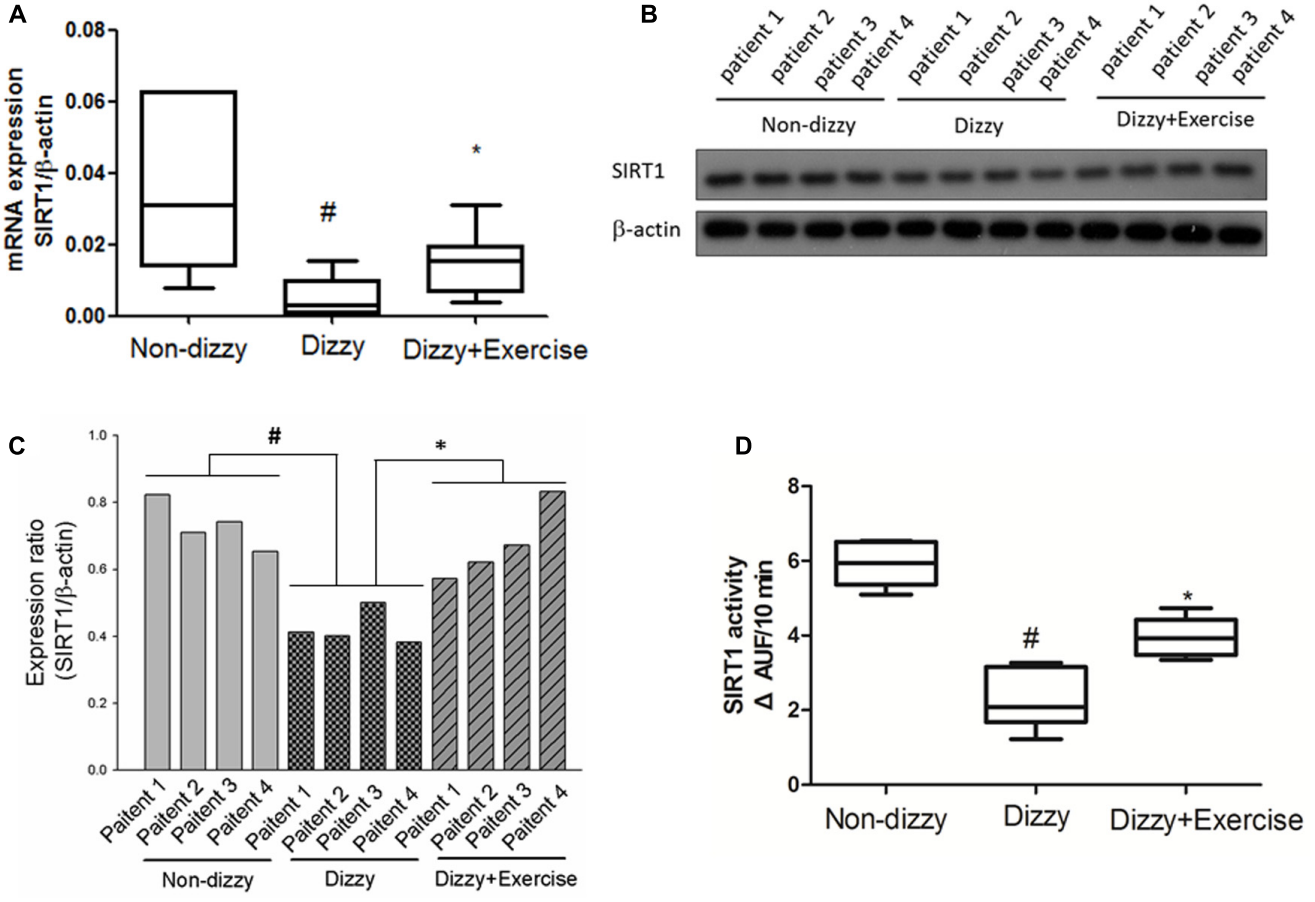
**Expression levels of SIRT1 in the blood samples of patients with dizziness before and after exercise training.** **(A)** Total blood mRNAs were extracted by kits, and the expression levels of SIRT1 in three different groups (non-dizzy, dizzy, and dizzy with exercise) were investigated using real-time PCR (each group, *n* = 15). **(B)** The samples of plasma were isolated from blood for Western blotting assay (each group, *n* = 4; from patient No. 1 to No. 4). **(C)** and the expression level were quantified as bar-chart. **(D)** The samples of plasma were isolated from blood for SIRT1 activity assay (non-dizzy, dizzy, and dizzy with exercise; each group, *n* = 15). Box-and-whisker plot illustrating the mean, standard deviation (S.D) and upper/lower extreme values for each group. ^#^*p* < 0.05 compared with the control group. *p < 0.05 compared with the dizzy group.

Our previous data indicated that exercise training improved the symptoms of dizziness (Kao et al., 2010b). However, the possible bio-molecular mechanism involved in dizzy patients is still largely unclear. SIRT1 is an important modulator in humans in the protection against oxidative events. A previous study reported that exercise alters SIRT1 expression levels in rats (Koltai et al., 2010). We hypothesize that the SIRT1 expression level as well as the catalytic activity of SIRT1 is suppressed in patients with chronic dizziness. To validate our hypothesis, the plasma samples obtained from the dizzy patients before exercise treatment and healthy donors (the control sample) were tested with the use of the real-time PCR, Western blotting, and the assay for detecting the deacetylase activity of SIRT1 (Kao et al., 2010a). Firstly, we showed that mRNA (**Figure 1A**) and protein (**Figure 1B**: Western blotting; **Figure 1C**: quantitative ratio for protein expression of Western blotting) expression levels of SIRT1 were significantly lower in patients with chronic dizziness compared with controls (*p* < 0.05). Moreover, the SIRT1 activity was repressed in the blood samples of chronic dizzy patients compared with those of non-dizzy group (*p* < 0.05; **Figure 1D**).

We further determined whether the vestibular exercise could affect the expression and enzymatic activity of SIRT1 in dizzy patients. Our results demonstrated that the mRNA (**Figure 1A**) and protein (**Figures 1B,C**) expression levels of SIRT1 in dizzy patients were significantly upregulated after exercise training (*p* < 0.05). The catalytic activities of SIRT1 in the blood samples of chronic dizzy patients increased significantly after vestibular training (**Figure 1D**), parallel to their symptomatic improvements (*p* < 0.05). These findings supported that exercise intervention not only reduced dizziness symptoms, but also improved SIRT1 functions in patients with chronic dizziness.

### THE EXPRESSION LEVELS OF SIRT1 DOWNSTREAM GENES WERE UP-REGULATED WITH EXERCISE TRAINING IN PATIENTS WITH CHRONIC DIZZINESS

**FIGURE 2:**
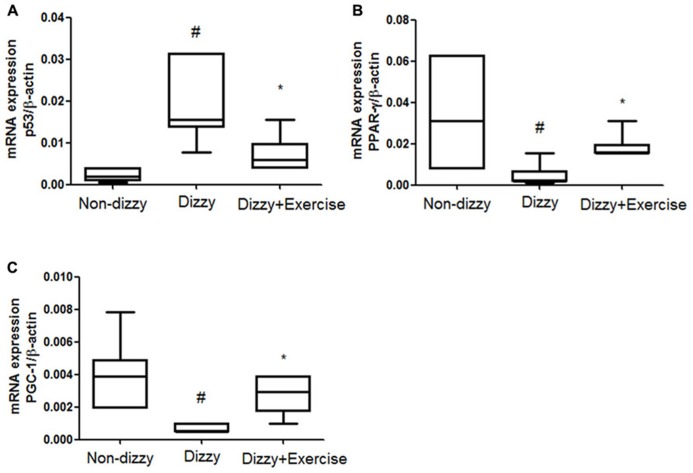
**Expression levels of SIRT1 downstream genes in the blood samples of patients with dizziness before or after exercise training.** Total blood mRNAs were isolated, and the **(A)** p53, **(B)** PPAR-γ, and **(C)** PGC-1 expression of the three different groups (non-dizzy, dizzy, and dizzy with exercise; each group, *n* = 15) were investigated using real-time PCR. Box-and-whisker plot illustrating the mean, standard deviation (S.D) and upper/lower extreme values for each group. ^#^*p* < 0.05 compared with the control group. *p < 0.05 compared with the dizzy group.

SIRT1 was previously reported to counteract the activation of p53 via deacetylating lysine 379 in response to genotoxic stress, thereby mitigating stress-induced cell death (Luo et al., 2001). Recent studies reported that p53 activation and the stress response function can be inhibited by SIRT1 (Luo et al., 2001; Langley et al., 2002). Our findings clearly demonstrated the protein levels and catalytic activities of SIRT1 in dizzy subjects were affected by vestibular training, we therefore went on to investigate the expression levels of p53 (an important downstream molecule of SIRT1) in non-dizzy and chronic dizzy patients before/after exercise training. As **Figure 2A** shows, using real-time PCR, we found that the p53 mRNA level was significantly higher in dizzy patients as compared with the level in non-dizzy subjects (*p* < 0.05; **Figure 2A**). Our experimental results also confirmed that p53 expression is mitigated with exercise training in patients with chronic dizziness (*p* < 0.05; **Figure 2A**).

In addition, two other important SIRT1-downstream target-genes, PPAR-γ coactivator-1α (PGC-1α) and peroxisome proliferator-activated receptor-gamma (PPAR-γ), were also evaluated in this study. We used real-time PCR to test whether the gene expression of PGC-1α and two other PPAR-γ were repressed and involved in chronic dizziness. The results, shown in **Figures 2B,C** indicated that the expression levels of blood PPAR-γ, PGC-1α were significantly diminished in patients with dizziness compared with controls (*p* < 0.05). In line with clinical symptomatic improvements, the expression levels of PPAR-γ and PGC-1α were remarkably increased in chronic dizzy patients after vestibular training (*p* < 0.05). Taken together, our findings indicate that exercise training enhances biological and physiological function involving the activation of SIRT1 axis and its downstream target-genes, including PGC-1α and PPAR-γ.

### THE EXPRESSION LEVELS OF FoxO FAMILY GENES AND ANTIOXIDANT ENZYME ACTIVITIES INCREASED WITH EXERCISE TRAINING IN PATIENTS WITH CHRONIC DIZZINESS

**FIGURE 3:**
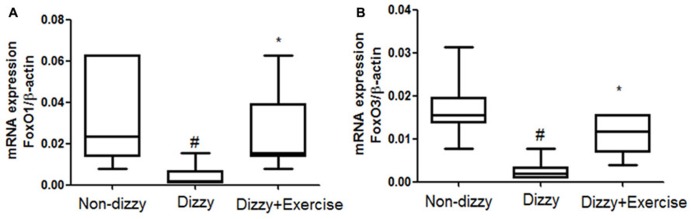
**Expression levels of FoxOs in the blood samples of patients with dizziness before or after exercise training.** Total blood mRNAs were isolated, and the **(A)** FoxO1 and **(B)** FoxO3 expression in three different groups (non-dizzy, dizzy, and dizzy with exercise; each group, *n* = 15) were investigated using real-time PCR. Box-and-whisker plot illustrating the mean, standard deviation (S.D) and upper/lower extreme values for each group. ^#^*p* < 0.05 compared with the control group. *p < 0.05 compared with the dizzy group.

FoxOs family has been reported to perform functions in modulating the bio-molecular processes, thereby regulating the responses for cellular oxidative stress (Hori et al., 2013). SIRT1 has been shown to deacetylate and activate three members of the FoxOs family (Brunet et al., 2004; Kobayashi et al., 2005). Recently, Hariharan et al. (2010) revealed that FoxO1 function is highly interrelated with SIRT1 activity. Brunet et al. (2004) further reported that SIRT1 may deacetylate FoxO3 under oxidative stress conditions, thus protecting against cellular stress resistance. FoxO1 and FoxO3 expression levels in our subjects were investigated to further determine whether the downstream genes of SIRT1 would be altered through vestibular training. Results showed that the mRNA expression levels of FoxO1 and FoxO3 in the blood samples of patients with dizziness were significantly diminished compared with those of controls (*p* < 0.05; **Figures 3A,B**). After exercise training, the mRNA expression levels of FoxO1 and FoxO3 were significantly upregulated in patients with dizzy subjects (*p* < 0.05; **Figures 3A,B**).

**FIGURE 4:**
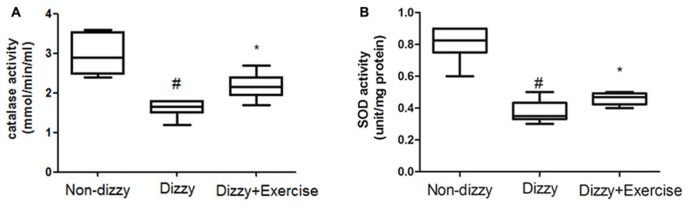
**Antioxidant enzyme activity in the blood samples of patients with dizziness before or after exercise training.** Plasma was isolated from whole blood. **(A)** Catalase and **(B)** SOD activity in three different groups (non-dizzy, dizzy, and dizzy with exercise; each group, *n* = 15) were investigated using kits. Box-and-whisker plot illustrating the mean, standard deviation (S.D) and upper/lower extreme values for each group. ^#^*p* < 0.05 compared with the control group. *p < 0.05 compared with the dizzy group.

It is now well established that cells activate SIRT1 and FoxOs transcription factors to reduce the level of oxidative stress via the induction of enzymes such as superoxide dismutase (SOD) and catalase (Kops et al., 2002; Nemoto and Finkel, 2002). For example, FoxO3 protects quiescent cells from oxidative stress by directly increasing the messenger RNA and protein quantities of SOD (Kops et al., 2002). We thus isolated the plasma from whole blood and tested the antioxidant enzymes activities in all subjects. As presented **Figures 4A,B**, we found that the activities of SOD and catalase were significantly reduced in patients with dizziness (*p* < 0.05, respectively). After exercise intervention, the activities of these antioxidant enzymes, SOD and catalase were significantly enhanced (*p* < 0.05; **Figures 4A,B**). Consistent with the increased expression of the FoxOs family and SIRT1-axis genes after treatment in dizzy patients, our findings supported that vestibular training may be conducive for activating antioxidant enzyme activities.

### OXIDATIVE STRESS WERE REVERSED WITH EXERCISE TRAINING IN PATIENTS WITH CHRONIC DIZZINESS

**FIGURE 5:**
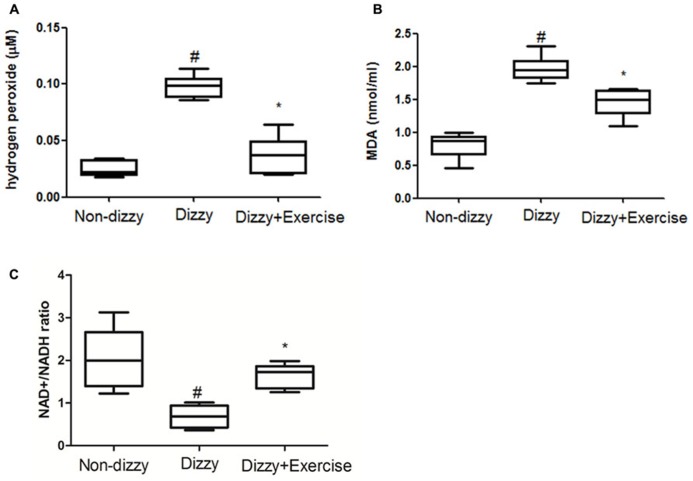
**ROS, MDA and NAD+/NADH ratio concentrations in the blood samples of patients with dizziness before or after exercise training.** Plasma was isolated from whole blood. **(A)** Hydrogen peroxide (H_2_O_2_), **(B)** malondialdehyde (MDA) concentration and **(C)** NAD+/NADH ratio in three different groups (non-dizzy, dizzy, and dizzy with exercise; each group, *n* = 15) were investigated using kits. Box-and-whisker plot illustrating the mean, standard deviation (S.D) and upper/lower extreme values for each group. ^#^*p* < 0.05 compared with the control group. *p < 0.05 compared with the dizzy group.

The relationship between ROS and dizziness has been studied (Semaan et al., 2005). Semaan et al. (2005) have found that patients who suffered from dizziness showed higher systemic oxidative stress compared with control groups (Calabrese et al., 2010). We confirmed that the concentration levels of ROS content, the hydrogen peroxide, in blood samples of patients with chronic dizziness were significantly higher than those in the non-dizzy individuals (*p* < 0.05; **Figure 5A**). We investigated the serum level of an oxidative parameter, malondialdehyde (MDA), to examine the oxidative status in patients with chronic dizziness. As presented in **Figure 5B**, the result showed that subjects with chronic dizziness had a higher MDA concentration as compared with those in the control group (*p* < 0.05). Notably, both the concentration levels of hydrogen peroxide and the MDA were significantly reduced after exercise training (*p* < 0.05, respectively).

SIRT1 is an NAD+-dependent protein deacetylase and exhibits the function to decrease ROS levels and participates in cell survival under oxidative stress conditions (Ou et al., 2014). It has been suggested that NAD+/NADP ratio is reduced under oxidative stimulation (Shetty et al., 2014). Based on these previous reports, we investigated the NAD+/NADP ratio in the peripheral blood samples of dizzy patients to verify whether NAD+/NADP ratio would be reduced under oxidative stimulation. As presented in **Figure 5C**, we found that patients with chronic dizziness indeed had a lower NAD+/NADP ratio compared with the control group (*p* < 0.05; **Figure 5C**). In contrast, the NAD+/NADP ratio in the blood samples of patients with chronic dizziness significantly increased after vestibular training (*p* < 0.05; **Figure 5C**). Our findings demonstrated that exercise training enhances NAD+/NADP activity. Vestibular rehabilitation could attenuate the blood ROS level, thus possibly contribute to a protective effect against oxidative stress in chronically dizzy patients.

## DISCUSSION

**FIGURE 6:**
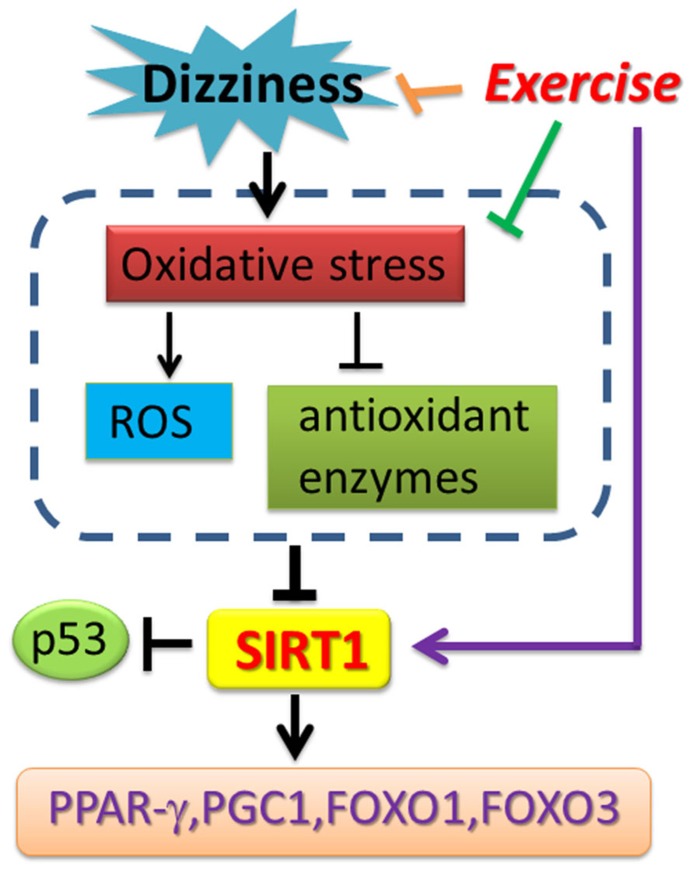
**Schematic diagram showing the potential mechanism of exercise training-derived SIRT1-related axis in patients with dizziness.** As depicted, exercise training reverses SIRT1 expression and also upregulates PPAR-γ, PGC-1 expression. Exercise training enhances antioxidant enzyme activity and reduces the ROS and MDA concentration in blood. The arrow (→) indicates activation or induction, and ⊣ indicates inhibition or blockade.

Chronic dizziness is a complex mixture of feelings including unsteadiness, lightheadedness and even walking difficulties (Colledge et al., 1994). In the aged population, dizziness can be a result of reduced visual acuity, disrupted vestibular reflexes, decreased somatosensory reactions, and declined musculoskeletal function (Herdman, 2007). Vestibular rehabilitation is a valuable treatment approach for patients with dizziness. Despite the fact that major previous studies investigated the relationship between SIRT1 expression change and skeletal muscles exercise training, vestibular rehabilitation involves both central nervous system compensation as well as muscle balance training. It is reasonable to believe that SIRT1 expression could be enhanced through a comprehensive, eye-head-trunk coordination training program. Recent clinical observations revealed that SIRT1 expression in peripheral blood mononuclear cells (PBMCs) from metabolic syndrome and insulin resistant subjects was significantly reduced compared with controls (de Kreutzenberg et al., 2010). The over-accumulated ROSs, including those generated from Glucotoxicity-, lypotoxicity-, senescence-induced oxidative stress, have been suggested to be one major risk in suppressing SIRT1 expression (de Kreutzenberg et al., 2010; Lin et al., 2011; Pardo and Boriek, 2011). The clinical relevance of SIRT1 expression and chronic dizziness has not yet been explored. To the best of our knowledge, this is the first study demonstrating that the mRNA and protein expression levels of SIRT1, as well as SIRT1 activity, were repressed in patients with chronic dizziness. We also proved that vestibular exercise training provides a non-invasive, non-pharmacological avenue to increase the blood SIRT1 expression in chronically dizzy patients. Exercise intervention also functionally inhibits p53 expression and enhances PGC-1α, PPAR-γ, and FoxOs expressions, thereby suppressing oxidative stress in patients with chronic dizziness. Based on our clinical findings and previous research reports, the possible bio-molecular mechanism involved in chronic dizzy is proposed in **Figure 6**.

The prevalence and the severity of dizziness increases with age (Colledge et al., 1994). The relationship between oxidative stress and dizziness has also been investigated in chronic dizziness (Semaan et al., 2005). Numerous studies have explored the relationship between SIRT1 expression and aging (Guarente, 2013; Xiong et al., 2013; Hubbard and Sinclair, 2014). SIRT1 has been reported to play a central role in the regulation of chronic pathogenesis, such as diabetes, neurodegenerative, and chronic inflammatory diseases (Bitterman et al., 2002; Duan, 2013; Min et al., 2013). FoxOs signaling is highly correlated with the control of immune system homeostasis in mammals (Lin et al., 2004). Lin et al. (2004) demonstrated the relationship between FoxO3 and inflammation, suggesting that multisystem inflammation may be caused by the lack of FoxO3a inhibition of NF-kB activities. It has been shown that SIRT1 could negatively regulate NF-κB expression and strongly suppressed the activation of pro-inflammatory genes in SIRT1-knock mice model (Schug et al., 2010). Recently, Zeng et al. (2013) reported that activation of SIRT1 inhibits LXRα expression, thereby inhibiting NF-kB activity. One previous report also suggested that the expression and nuclear translocation of FOXO3a is enhanced after resveratrol pretreatment because FOXO3a is a transcription factor that activates the human MsrA promoter (Wu et al., 2013). The above mentioned investigations concluded that enhancing SIRT1 function may have a role in effectively inhibiting pro-inflammatory responses, possibly, through modulating the FoxOs family derived signaling. In the present study, we found that dizzy patients had lower FoxO1 and FoxO3 expression levels. Together with the downregulated SIRT1 expression/activity, our data indicate that chronic dizzy patients might be constantly under the influence of chronic inflammation, that leads to uncomfortable symptoms.

Clinical treatment using radical scavengers, such as rebamipide, vitamin C and glutathione, has made significant improvement of vertigo, indicating the potential of these scavengers as effective therapeutics for chronic dizziness (Raponi et al., 2003; Takumida et al., 2003). FoxO3a protects quiescent cells from oxidative stress by directly increasing their quantities of manganese superoxide dismutase (MnSOD) messenger RNA and protein. FOXO3 directly binds to an MnSOD promoter at FOXO binding elements to increase its expression. The activation of MnSOD in mitochondria protects cells from ROS-mediated damage (Kops et al., 2002). In accordance with previous findings, we demonstrated that exercise training could effectively enhance FoxO1/FoxO3 expression (**Figures 3A,B**), remarkably upregulate the antioxidant-enzyme of SOD and catalase (**Figures 4A,B**), and significantly suppress the content of ROS-oxidative stress in dizzy patients (**Figures 5A,B**). These results further suggest that effective vestibular training could alleviate the status of chronic inflammation and ROS-induced oxidative stress in chronic dizziness, possibly through the activation of FoxOs family genes and SIRT1-related axis.

Physical exercise intervention has been recognized as an effective treatment strategy for the management of symptoms associated with vestibular dysfunction that manifests as dizziness and imbalance related to body position or movement in patients with dizziness (Porciuncula et al., 2012). Falone et al. (2012) showed that exercise training enhances human SIRT1 expression in the hippocampus, and Lezi et al. reported that exercise training upregulates SIRT1, AMPK and PGC-1 function in the liver and brain (Lezi et al., 2013). Because the mechanisms of vestibular rehabilitation involves adaptation through central nervous system compensation (Herdman, 2007), and based on these studies, we hypothesize that exercise-induced protection effects might be derived through the modulation of SIRT1 and PGC-1α axis. In this present study, our data showed that the expression level of SIRT1, as well as PGC-1 and PPAR-γ are reversed by exercise training in patients with chronic dizziness. Accumulative evidence in our results indicate that exercise training enhances biological function and physiological function involving the activation of SIRT1 axis and its downstream target-genes.

The limitation of our study is that this is a cross-sectional design. A longitudinal study would be valuable to determine the long term effects of vestibular training. Furthermore, SIRT1 is known to regulate other anti-inflammation kinases, such as AMPK or AKT. Future research may need to include kinase activity assays or Western blotting for pro-inflammatory proteins to further validate the roles of other anti-inflammation kinase activities.

## CONCLUSION

In this study, we confirmed that the blood SIRT1 level is attenuated in patients with chronic dizziness. Exercise training was found to correct dizziness symptoms and, at the same time, reverse SIRT1 expression and activity in blood. These novel findings may shed light on a new therapeutic strategy for managing chronic dizziness, especially for targeting the SIRT1-specific axis.

## Conflict of Interest Statement

The authors declare that the research was conducted in the absence of any commercial or financial relationships that could be construed as a potential conflict of interest.

## References

[B1] Bisdorff A. Von Brevern M. Lempert T. Newman-Toker D. E. (2009). Classification of vestibular symptoms: towards an international classification of vestibular disorders. *J. Vestib. Res.* 19 1–13 10.3233/VES-2009-0343 19893191

[B2] Bitterman K. J. Anderson R. M. Cohen H. Y. Latorre-Esteves M. Sinclair D. A. (2002). Inhibition of silencing and accelerated aging by nicotinamide, a putative negative regulator of yeast sir2 and human SIRT1. *J. Biol. Chem.* 277 45099–45107 10.1074/jbc.M205670200 12297502

[B3] Boyer F. C. Percebois-Macadre L. Regrain E. Leveque M. Taiar R. Seidermann L. (2008). Vestibular rehabilitation therapy. *Neurophysiol. Clin.* 38 479–487 10.1016/j.neucli.2008.09.011 19026967

[B4] Brunet A. Sweeney L. B. Sturgill J. F. Chua K. F. Greer P. L. Lin Y. (2004). Stress-dependent regulation of FOXO transcription factors by the SIRT1 deacetylase. *Science* 303 2011–2015 10.1126/science.1094637 14976264

[B5] Calabrese V. Cornelius C. Maiolino L. Luca M. Chiaramonte R. Toscano M. A. (2010). Oxidative stress, redox homeostasis and cellular stress response in Meniere's disease: role of vitagenes. *Neurochem. Res.* 35 2208–2217 10.1007/s11064-010-0304-2 21042850

[B6] Chen P. Y. Hsieh W. L. Wei S. H. Kao C. L. (2012). Interactive wiimote gaze stabilization exercise training system for patients with vestibular hypofunction. *J. Neuroeng. Rehabil*. 9 77 10.1186/1743-0003-9-77 PMC3481473 23043886

[B7] Colledge N. R. Wilson J. A. Macintyre C. C. MacLennan W. J. (1994). The prevalence and characteristics of dizziness in an elderly community. *Age Ageing* 23 117–120 10.1093/ageing/23.2.117 8023718

[B8] Csiszar A. Labinskyy N. Jimenez R. Pinto J. T. Ballabh P. Losonczy G. (2009). Anti-oxidative and anti-inflammatory vasoprotective effects of caloric restriction in aging: role of circulating factors and SIRT1. *Mech. Ageing Dev*. 130 518–527 10.1016/j.mad.2009.06.004 19549533 PMC2756526

[B9] De Angelis K. Schaan B. D. Maeda C. Y. Dall’Ago P. Wichi R. B. Irigoyen M. C. (2002). Cardiovascular control in experimental diabetes. *Braz. J. Med. Biol. Res.* 35 1091–1100 10.1590/S0100-879X2002000900010 12219181

[B10] de Kreutzenberg S. V. Ceolotto G. Papparella I. Bortoluzzi A. Semplicini A. Dalla Man C. (2010). Downregulation of the longevity-associated protein sirtuin 1 in insulin resistance and metabolic syndrome: potential biochemical mechanisms. *Diabetes* 59 1006–1015 10.2337/db09-1187 20068143 PMC2844808

[B11] Dieterich M. Eckhardt-Henn A. (2004). Neurological and somatoform vertigo syndromes. *Nervenarzt* 75 281–302 10.1007/s00115-003-1678-z 15074322

[B12] Dominguez M. O. (2005). Treatment and rehabilitation in vestibular neuritis. *Rev. Laryngol. Otol. Rhinol*. 126 283–286 16496560

[B13] Duan W. (2013). Sirtuins: from metabolic regulation to brain aging. *Front. Aging Neurosci.* 5:36 10.3389/fnagi.2013.00036 PMC3719022 23888142

[B14] Lezi E. Lu J. Burns J. M. Swerdlow R. H. (2013). Effect of exercise on mouse liver and brain bioenergetic infrastructures. *Exp. Physiol*. 98 207–219 10.1113/expphysiol.2012.066688 22613742 PMC3540163

[B15] Eaton D. A. Roland P. S. (2003). Dizziness in the older adult, Part 2. Treatments for causes of the four most common symptoms. *Geriatrics* 58 46 49–52 12708155

[B16] Eckhardt-Henn A. Best C. Bense S. Breuer P. Diener G. Tschan R. (2008). Psychiatric comorbidity in different organic vertigo syndromes. *J. Neurol*. 255 420–428 10.1007/s00415-008-0697-x 18338198

[B17] Falone S. D’Alessandro A. Mirabilio A. Cacchio M. Di Ilio C. Di Loreto S. (2012). Late-onset running biphasically improves redox balance, energy- and methylglyoxal-related status, as well as SIRT1 expression in mouse hippocampus. *PloS ONE* 7:e48334 10.1371/journal.pone.0048334 PMC3482192 23110231

[B18] Gao Z. Ye J. (2008). Inhibition of transcriptional activity of c-JUN by SIRT1. *Biochem. Biophys. Res. Commun*. 376 793–796 10.1016/j.bbrc.2008.09.079 18823944 PMC2629791

[B19] Ghosh S. Pulinilkunnil T. Yuen G. Kewalramani G. An D. Qi D. (2005). Cardiomyocyte apoptosis induced by short-term diabetes requires mitochondrial GSH depletion. *Am. J. Physiol. Heart Circ. Physiol*. 289 H768–H776 10.1152/ajpheart.00038.2005 15805231

[B20] Guarente L. (2013). Introduction: sirtuins in aging and diseases. *Methods Mol. Biol*. 1077 3–10 10.1007/978-1-62703-637-5_1 24014396

[B21] Hariharan N. Maejima Y. Nakae J. Paik J. Depinho R. A Sadoshima J. (2010). Deacetylation of FoxO by Sirt1 plays an essential role in mediating starvation-induced autophagy in cardiac myocytes. *Circ. Res.* 107 1470–1482 10.1161/CIRCRESAHA.110.227371 20947830 PMC3011986

[B22] Herdman S. J. (2007). *Vestibular Rehabilitation*. Philadelphia: F. A. Davis Company

[B23] Herdman S. J. Schubert M. C. Tusa R. J. (2001). Strategies for balance rehabilitation: fall risk and treatment. *Ann. N. Y. Acad. Sci.* 942 394–412 10.1111/j.1749-6632.2001.tb03762.x 11710480

[B24] Hori Y. S. Kuno A. Hosoda R. Horio Y. (2013). Regulation of FOXOs and p53 by SIRT1 modulators under oxidative stress. *PloS ONE* 8:e73875 10.1371/journal.pone.0073875 PMC3770600 24040102

[B25] Hubbard B. P. Sinclair D. A. (2014). Small molecule SIRT1 activators for the treatment of aging and age-related diseases. *Trends Pharmacol. Sci*. 10.1016/j.tips.2013.12.004 [Epub ahead of print] PMC3970218 24439680

[B26] Jacobson G. P. Newman C. W. (1990). The development of the Dizziness Handicap Inventory. *Arch. Otolaryngol. Head Neck Surg.* 116 424–427 10.1001/archotol.1990.01870040046011 2317323

[B27] Kang E. B. Kwon I. S. Koo J. H. Kim E. J. Kim C. H. Lee J. (2013). Treadmill exercise represses neuronal cell death and inflammation during Abeta-induced ER stress by regulating unfolded protein response in aged presenilin 2 mutant mice. *Apoptosis* 18 1332–1347 10.1007/s10495-013-0884-9 23907580

[B28] Kao C. L. Chen L. K. Chang Y. L. Yung M. C. Hsu C. C. Chen Y. C. (2010a). Resveratrol protects human endothelium from H(2)O(2)-induced oxidative stress and senescence via SirT1 activation. *J. Atheroscler. Thromb*. 17 970–979 10.5551/jat.4333 20644332

[B29] Kao C. L. Chen L. K. Chern C. M. Hsu L. C. Chen C. C. Hwang S. J. (2010b). Rehabilitation outcome in home-based versus supervised exercise programs for chronically dizzy patients. *Arch. Gerontol. Geriatr*. 51 264–267 10.1016/j.archger.2009.11.014 20022390

[B30] Kim S. E. Ko I. G. Shin M. S. Kim C. J. Jin B. K. Hong H. P. (2013). Treadmill exercise and wheel exercise enhance expressions of neutrophic factors in the hippocampus of lipopolysaccharide-injected rats. *Neurosci. Lett*. 538 54–59 10.1016/j.neulet.2013.01.039 23403101

[B31] Kobayashi Y. Furukawa-Hibi Y. Chen C. Horio Y. Isobe K. Ikeda K. (2005). SIRT1 is critical regulator of FOXO-mediated transcription in response to oxidative stress. *Int. J. Mol. Med.* 16 237–243 16012755

[B32] Koltai E. Szabo Z. Atalay M. Boldogh I. Naito H. Goto S. (2010). Exercise alters SIRT1, SIRT6, NAD and NAMPT levels in skeletal muscle of aged rats. *Mech. Ageing Dev.* 131 21–28 10.1016/j.mad.2009.11.002 19913571 PMC2872991

[B33] Kops G. J. Dansen T. B. Polderman P. E. Saarloos I. Wirtz K. W. Coffer P. J. (2002). Forkhead transcription factor FOXO3a protects quiescent cells from oxidative stress. *Nature* 419 316–321 10.1038/nature01036 12239572

[B34] Langley E. Pearson M. Faretta M. Bauer U. M. Frye R. A. Minucci S. (2002). Human SIR2 deacetylates p53 and antagonizes PML/p53-induced cellular senescence. *EMBO J*. 21 2383–2396 10.1093/emboj/21.10.2383 12006491 PMC126010

[B35] Lavu S. Boss O. Elliott P. J. Lambert P. D. (2008). Sirtuins – novel therapeutic targets to treat age-associated diseases. *Nat. Rev. Drug Discov*. 7 841–853 10.1038/nrd2665 18827827

[B36] Lezi E. Lu J. Burns J. M. Swerdlow R. H. (2013). Effect of exercise on mouse liver and brain bioenergetic infrastructures. *Exp. physiol.* 98 207–219 10.1113/expphysiol.2012.066688 22613742 PMC3540163

[B37] Lin L. Hron J. D. Peng S. L. (2004). Regulation of NF-kappaB, Th activation, and autoinflammation by the forkhead transcription factor Foxo3a. *Immunity* 21 203–213 10.1016/j.immuni.2004.06.016 15308101

[B38] Lin T. J. Peng C. H. Chiou S. H. Liu J. H. Lin Chung W. Tsai C. Y. (2011). Severity of lens opacity, age, and correlation of the level of silent information regulator T1 expression in age-related cataract. *J. Cataract Refract. Surg*. 37 1270–1274 10.1016/j.jcrs.2011.02.027 21700104

[B39] Ljubicic V. Khogali S. Renaud J. M. Jasmin B. J. (2012). Chronic AMPK stimulation attenuates adaptive signaling in dystrophic skeletal muscle. *Am. J. Physiol. Cell Physiol*. 302 C110–C121 10.1152/ajpcell.00183.2011 21940670

[B40] Luo J. Nikolaev A. Y. Imai S. Chen D. Su F. Shiloh A. (2001). Negative control of p53 by Sir2alpha promotes cell survival under stress. *Cell* 107 137–148 10.1016/S0092-8674(01)00524-4 11672522

[B41] Luxon L. M. (2004). Evaluation and management of the dizzy patient. *J. Neurol. Neurosurg. Psychiatry* 75(Suppl 4) iv45–52 10.1136/jnnp.2004.055285 15564431 PMC1765678

[B42] Milne J. C. Denu J. M. (2008). The Sirtuin family: therapeutic targets to treat diseases of aging. *Curr. Opin. Chem. Biol*. 12 11–17 10.1016/j.cbpa.2008.01.019 18282481

[B43] Min S. W. Sohn P. D. Cho S. H. Swanson R. A. Gan L. (2013). Sirtuins in neurodegenerative diseases: an update on potential mechanisms. *Front. Aging Neurosci*. 5:53 10.3389/fnagi.2013.00053 PMC3782645 24093018

[B44] Moreau M. Neveu M. Stephan S. Noblesse E. Nizard C. Sadick N. S. (2007). Enhancing cell longevity for cosmetic application: a complementary approach. *J. Drugs Dermatol*. 6 s14–s19 17691205

[B45] Nemoto S. Finkel T. (2002). Redox regulation of forkhead proteins through a p66shc-dependent signaling pathway. *Science* 295 2450–2452 10.1126/science.1069004 11884717

[B46] Niehaus W. G. Jr. Samuelsson B. (1968). Formation of malonaldehyde from phospholipid arachidonate during microsomal lipid peroxidation. *Eur. J. Biochem.* 6 126–130 10.1111/j.1432-1033.1968.tb00428.x 4387188

[B47] Ou X. Lee M. R. Huang X. Messina-Graham S. Broxmeyer H. E. (2014). SIRT1 positively regulates autophagy and mitochondria function in embryonic stem cells under oxidative stress. *Stem Cells* 10.1002/stem.1641 [Epub ahead of print] PMC3991763 24449278

[B48] Paparella M. M. Alleva M. Bequer N. G. (1990). Dizziness. *Primary Care* 17 299–308 2196611

[B49] Pardo P. S. Boriek A. M. (2011). The physiological roles of Sirt1 in skeletal muscle. *Aging* 3 430–437 21483036 10.18632/aging.100312 PMC3117458

[B50] Porciuncula F. Johnson C. C. Glickman L. B. (2012). The effect of vestibular rehabilitation on adults with bilateral vestibular hypofunction: a systematic review. *J. Vestib. Res.* 22 283–298 10.3233/VES-120464 23302709

[B51] Raponi G. Alpini D. Volonte S. Capobianco S. Cesarani A. (2003). The role of free radicals and plasmatic antioxidant in Meniere's syndrome. *Int. Tinnitus J.* 9 104–108 15106283

[B52] Salminen A. Kaarniranta K. (2010). Insulin/IGF-1 paradox of aging: regulation via AKT/IKK/NF-kappaB signaling. *Cell. Signal*. 22 573–577 10.1016/j.cellsig.2009.10.006 19861158

[B53] Sauve A. A. Wolberger C. Schramm V. L. Boeke J. D. (2006). The biochemistry of sirtuins. *Annu. Rev. Biochem*. 75 435–465 10.1146/annurev.biochem.74.082803.133500 16756498

[B54] Schubert M. C. Minor L. B. (2004). Vestibulo-ocular physiology underlying vestibular hypofunction. *Phys. Therapy* 84 373–385 15049730

[B55] Schug T. T. Xu Q. Gao H. Peres-da-Silva A. Draper D. W. Fessler M. B. (2010). Myeloid deletion of SIRT1 induces inflammatory signaling in response to environmental stress. *Mol. Cell. Biol*. 30 4712–4721 10.1128/MCB.00657-10 20647536 PMC2950528

[B56] Semaan M. T. Alagramam K. N. Megerian C. A. (2005). The basic science of Meniere's disease and endolymphatic hydrops. *Curr. Opin. Otolaryngol. Head Neck Surg*. 13 301–307 10.1097/01.moo.0000186335.44206.1c 16160525

[B57] Shetty P. K. Galeffi F. Turner D. A. (2014). Nicotinamide pre-treatment ameliorates NAD(H) hyperoxidation and improves neuronal function after severe hypoxia. *Neurobiol. Dis*. 62 469–478 10.1016/j.nbd.2013.10.025 24184921 PMC4143422

[B58] Takumida M. Anniko M. Ohtani M. (2003). Radical scavengers for Meniere's disease after failure of conventional therapy: a pilot study. *Acta Otolaryngol*. 123 697–703 12953767 10.1080/00016480310000728a

[B59] Telian S. A. Shepard N. T. (1996). Update on vestibular rehabilitation therapy. *Otolaryngol. Clin. North Am*. 29 359–371 8860934

[B60] Tinetti M. E. (1986). Performance-oriented assessment of mobility problems in elderly patients. *J. Am. Geriatr. Soc*. 34 119–126 3944402 10.1111/j.1532-5415.1986.tb05480.x

[B61] Williams C. B. Gurd B. J. (2012). Skeletal muscle SIRT1 and the genetics of metabolic health: therapeutic activation by pharmaceuticals and exercise. *Appl. Clin. Genet*. 5 81–91 10.2147/TACG.S31276 23776383 PMC3681195

[B62] Wu P. F. Xie N. Zhang J. J. Guan X. L. Zhou J. Long L. H. (2013). Resveratrol preconditioning increases methionine sulfoxide reductases A expression and enhances resistance of human neuroblastoma cells to neurotoxins. *J. Nutr. Biochem*. 24 1070–1077 10.1016/j.jnutbio.2012.08.005 23022493

[B63] Xiong H. Dai M. Ou Y. Pang J. Yang H. Huang Q. (2013). SIRT1 expression in the cochlea and auditory cortex of a mouse model of age-related hearing loss. *Exp. Gerontol*. 51C 8–14 10.1016/j.exger.2013.12.006. 24365660

[B64] Yeung F. Hoberg J. E. Ramsey C. S. Keller M. D. Jones D. R. Frye R. A. (2004). Modulation of NF-kappaB-dependent transcription and cell survival by the SIRT1 deacetylase. *EMBO J*. 23 2369–2380 10.1038/sj.emboj.7600244 15152190 PMC423286

[B65] Yoshizaki T. Schenk S. Imamura T. Babendure J. L. Sonoda N. Bae E. J. (2010). SIRT1 inhibits inflammatory pathways in macrophages and modulates insulin sensitivity. *Am. J. Physiol. Endocrinol. Metab*. 298 E419–E428 10.1152/ajpendo.00417.2009 19996381 PMC2838524

[B66] Zeng H. T. Fu Y. C. Yu W. Lin J. M. Zhou L. Liu L. (2013). SIRT1 prevents atherosclerosis via liverXreceptor and NFkappaB signaling in a U937 cell model. *Mol. Med. Rep*. 8 23–28 10.3892/mmr.2013.1460 23652462

